# Carnosine Stimulates Macrophage-Mediated Clearance of Senescent Skin Cells Through Activation of the AKT2 Signaling Pathway by CD36 and RAGE

**DOI:** 10.3389/fphar.2020.593832

**Published:** 2020-12-16

**Authors:** Xuenan Li, Kaiye Yang, Shuang Gao, Jungang Zhao, Guangrong Liu, Yu Chen, Haojie Lin, Wengang Zhao, Zhenlin Hu, Nuo Xu

**Affiliations:** ^1^ College of Life and Environmental Sciences, Wenzhou University, Wenzhou, China; ^2^ Infinitus (China) Company Ltd., Guangzhou, China; ^3^ School of Pharmaceutical Sciences, Wenzhou Medical University, Wenzhou, China

**Keywords:** Akt2, senescent cell, macrophages, carnosine, skin aging, co-culture

## Abstract

**Background:** Macrophages can selectively recognize and eliminate senescent cells, but this function is impaired with age, resulting in excessive accumulation of senescent cells in the skin, which ultimately causes skin aging. Therefore, enhancing the immune surveillance ability of macrophages to clear senescent keratinocytes and fibroblasts from aging skin may be an effective skin rejuvenation strategy.

**Methods:** In this study, a macrophage and senescent skin cell co-culture model was established whereby THP-1-derived macrophages and tert-butyl hydroxide-induced senescent skin cells (HaCaT and HFF-1) were grown in the same culture. Senescent skin cells were detected by the SPiDER-βgal assay, and the expression of secretory phenotype factors related to senescence was assayed by qPCR. The effect of carnosine on the number of SA-β-gal positive skin cells in the macrophage-senescent skin cell co-culture was evaluated and compared with that in the senescent skin cell monoculture.

**Results:** Carnosine promoted macrophage-mediated elimination of senescent skin cells in the co-culture. Through the AKT2 signaling pathway, carnosine upregulated the expression of CD36 and receptors for advanced glycation end products and elevated the phagocytic capacity of the macrophages, thereby promoting the ability of the macrophages to eliminate the senescent skin cells.

**Conclusions:** Carnosine could boost the immune surveillance ability of macrophages to clear senescent keratinocytes and fibroblasts in the macrophage-senescent skin cell co-culture by activating the AKT2 signaling pathway, suggesting the possibility of using carnosine as an agent to reverse skin aging.

## Introduction

The skin of an animal lies at the interface between the internal environment of the animal and the outside environment where it serves as a protective barrier, providing protection against microorganisms and preserving the fluid and temperature homeostasis. The skin is the largest body organ, and increased wrinkles, sagging and laxity are the most visible and obvious manifestations of the skin that come with aging (Baumann, 2007). Effective interventions are required to address the rise in the aging population in our society and the psychosocial impact of skin aging, which can be rather dramatic. It is worth noting that although some treatments such as retinoids, antioxidants, hormone replacement therapy and stem cell therapy may help to delay skin aging (McCullough and Kelly, 2006; Zhang and Duan, 2018; Chaudhary et al., 2020), a more rigorous approach is required to efficiently prevent the occurrence of skin aging. Keratinocytes as well as fibroblasts play a critical part in skin aging and they are also the cell types most studied in the investigation of the senescence process. Previous studies have revealed that accumulation of senescent keratinocytes and fibroblasts found in aged skin may drive the process of skin aging, which is specifically characterized by functional deterioration (van Deursen, 2014; Gruber et al., 2020).

Cellular senescence is considered as the main effector and a common element in aging. A hallmark of senescent cells is the secretion of factors associated with the senescence-associated secretory phenotype (SASP), which include chemokines (e.g., CXCR2), inflammatory cytokines (e.g., interleukin-6 and interleukin-8), growth factors and matrix metalloproteinases (MMPs) (Ghosh and Capell, 2016; Wang and Dreesen, 2018). The incessant secretion of SASP factors keeps the skin tissue in a chronic inflammatory state and alters the tissue microenvironment. MMPs can degrade collagen and other extracellular matrix components in the dermal connective tissue, as well as diminishing the elasticity of the skin (Fisher et al., 2002; Freund et al., 2010; Ghosh and Capell, 2016; Fussell and Kelly, 2020).

The selective elimination of senescent cells once they appear has turned out to be an effective therapy to reverse aging (McHugh and Gil, 2018). As demonstrated in naturally aged and fast-aging XpdTTD/TTD mice, senescence accompanied by p53 nuclear exclusion followed by apoptosis of the senescent cells can restore fur density, fitness and renal function (Baar et al., 2017). A number of studies have found that the elimination of senescent cells is primarily regulated by immunosurveillance (Pérez-Mancera et al., 2014), but the underlying mechanism remains poorly understood. The ability of immune cells to recognize and remove senescent cells is impaired with age, consequently causing the senescent cells to accumulate in the tissues. This impairment of immune function has been termed immunosenescence (Ovadya and Krizhanovsky, 2014; Jeon et al., 2017; Burton and Stolzing, 2018). Macrophages are involved in the critical immune surveillance of senescent cells. Although the mechanisms are uncertain, the depletion of macrophages in mice can greatly reduce the immune surveillance of senescent cells (Yun et al., 2015; Hall et al., 2016). Therefore, boosting the immune surveillance ability of macrophages to clear senescent keratinocytes and fibroblasts in aged skin may be an effective strategy for skin rejuvenation (Toutfaire et al., 2017).

Carnosine is an endogenous dipeptide consisting of L-histidine with β-alanine. It is synthesized by the enzyme carnosine synthase. Carnosine has been widely used as a topical agent for the treatment of skin and clinical studies have shown that skin treated with facial cream formulated with carnosine can become visibly tightened, more elastic and less dry (Garre et al., 2017; Guaitolini et al., 2019). Carnosine has protective functions in addition to its anti-oxidant and free-radical scavenging roles. It can extend the life span of cultured human fibroblasts, inhibit protein glycation (formation of cross-links, carbonyl groups and AGEs) and delay aging in senescence-accelerated mice (Hipkiss, 1998; Boldyrev et al., 1999; Gallant et al., 2000; Shao et al., 2004). The anti-aging mechanisms of carnosine involve the inhibition of the mTOR and TGF/Smad3 pathways, antioxidation as well as antiglycation (Hobart et al. 2004; Reddy et al., 2005; Hipkiss et al., 2016). Carnosine can modulate the immune response in human neutrophils as well as increasing the phagocytic activity of peritoneal macrophage (Onufriev et al., 1992; Tan and Candlish, 1998). The ability of carnosine to modulate the production of NO and the polarization of macrophage RAW 264.7 has also been demonstrated (Caruso et al., 2017; Caruso et al., 2019). Zinc L-carnosine (ZnC) has also been reported to suppress LPS-induced inflammatory through activating the Nrf2/HO-1 or inhibiting the nuclear factor-kappaB signaling pathway in RAW 264.7 cells (Ooi et al., 2016; Ooi et al., 2017).

In this study, we explored the effect of carnosine on senescent keratinocytes and fibroblasts and established a co-culture model consisting of macrophages and senescent cells to determine whether carnosine could stimulate the clearance of senescent cells mediated by macrophage.

## Materials and Methods

### Reagents

Carnosine (purity: 99.6%) was supplied by Winkey Pharmaceutical (Shenzhen, China). Tert-butyl hydroperoxide (t-BHP) solution and phorbol 12-myristate 13-acetate (PMA) were purchased from Macklin (Shanghai, China). Azeliragon and CCT128930 were purchased from Selleck (Shanghai, China). Sulfosuccinimidyl oleate was obtained from MCE (Shanghai, China).

### Cell Cultures

Immortalized human keratinocyte (HaCaT) and human foreskin fibroblast (HFF-1) were supplied by Zhong Qiao Xin Zhou Biotechnology Co., Ltd. (Shanghai, China). HaCaT and HFF-1 cells were cultured in Dulbecco’s modified Eagle’s medium (DMEM) (GIBCO, Life Technologies Corporation, NY, United States) containing 10% fetal bovine serum (FBS) (GIBCO, Life Technologies Corporation, NY, United States) at 37°C in a humidified atmosphere containing 5% CO_2_. Human monocyte leukemia cell line, THP-1, was supplied by the Cell Bank of Typical Culture Preservation Committee of Chinese Academy of Science (Shanghai, China). THP-1 cells were cultured in RPMI medium 1640 (GIBCO, Life Technologies Corporation, NY, United States) containing 10% FBS at 37°C in a humidified atmosphere containing 5% CO_2_. Macrophages (Mφ) were acquired from THP-1 cells by induction with 200 ng/ml PMA for 6 h as previously described (Tsai et al., 2017; Kaneko et al., 2018; Kurynina et al., 2018) but with minor modification.

### Cell Viability Assay

THP-1 cells cultured in 96-well plates at a density of 2 × 10^4^/well were induced with PMA for 6 h followed by treatment with the indicated concentrations of carnosine (5, 10, 30 mM) for 24 h and then subjected to cell viability assay performed with Cell Counting Kit-8 (CCK-8) (Dojindo Laboratories, Japan) according to the manufacturer's instruction.

### Senescence Associated β-Galactosidase Staining

Tert-butyl hydroperoxide was used to induce the onset of senescence in HaCaT and HFF-1 cells. β-Galactosidase (SA-β-gal) positive cells associated with senescence were detected using SPiDER-β Gal Kit (DOJINDO, Tokyo, Japan). In brief, the cells were first fixed with 4% paraformaldehyde for 3 min at 25°C and then rinsed three times with HBSS solution. This was followed by incubation with 200 μL of fresh staining solution of SA-β-Gal for 15 min at 37°C. SA-β-gal activity measured under each condition was expressed as the percentage of positive cells. The SA-β-gal positive cells were monitored with a fluorescence microscope (Nikon, Tokyo, Japan) using *λ*_Em_ and *λ*_Ex_ of 516 and 494 nm, respectively.

### Quantitative Real-Time PCR (*q*RT-PCR)

HaCaT cells were incubated with tBHP at different concentrations (0, 150, 200, 250, and 300 μM) for 2 h, followed by incubation without t-BHP for 12 h. Interleukin 6 (IL-6) and interleukin 8 (IL-8) mRNA levels in the HaCaT cells were then assayed by *q*RT-PCR. HFF-1 cells were also treated with t-BHP at different concentrations (0, 100, 200, 300, and 400 μM) for 2 h, followed by incubation without t-BHP for 12 h. The mRNA levels of MMP-1 and MMP-3 in the HFF-1 cells were assayed by *q*RT-PCR. HaCaT cells were pretreated with tBHP (250 μM) and cultured for an additional 12 h without tBHP to induce the onset of senescence, followed by exposure to carnosine for another 24 h. After that, the levels of IL-6 and IL-8 mRNA were assayed by *q*RT-PCR. HFF-1 cells were also pretreated with 300 μM of tBHP for 2 h and the medium was replaced with fresh DMEM followed further incubation for 12 h before exposure to carnosine for 24 h. Following carnosine exposure, the mRNA levels of MMP-1 and MMP-3 in the cells were assayed by *q*RT-PCR. In brief, an RNA isolation kit (Biomiga, San Diego, CA, United States) was used to extract the total RNA according to the manufacturer’s instructions. The concentration of total RNA obtained was determined using a NanoQuant Plate (Tecan, Mannedorf, Switzerland). Reverse transcription was performed with 1 μg of total RNA using PrimeScript RT reagent Kit (Takara, Dalian, China). *q*RT-PCR was performed with SYBR Green Master Mix (Applied Biosystems, Foster City, CA) using the LC96 system (Roche, Basel, Switzerland). The primers sequences are shown in Table 1. The expression of target genes was normalized to the level of GAPDH, which served as an endogenous control. The relative expression of each targeted gene was analyzed using the 2^−ΔΔCt^ method.

**TABLE 1:** The primers sequences for quantitative PCR (qPCR).

Gene	Primer	Primer sequence (5′–3′)
IL-6	Forward	CCGAAGGACGGGAGCAG
	Reverse	GGG​TCA​GGG​GTG​GTT​ATT​GC
IL-8	Forward	AGCAGTTCCACAGGCACA
	Reverse	ACT​CTG​GTT​GGC​TTC​CTT​CA
MMP-1	Forward	TGG​GCT​GAA​AGT​GAC​TGG​GAA​AC
	Reverse	ACA​TCT​GGG​CTG​CTT​CAT​CAC​C
MMP-3	Forward	AGT​TCC​TTG​GAT​TGG​AGG​TGA​CG
	Reverse	TTC​GGG​ATG​CCA​GGA​AAG​GTT​C
CD14	Forward	ACT​CCC​TCA​ATC​TGT​CGT​TCG​C
	Reverse	AGC​TTG​GCT​GGC​AGT​CCT​TTA​G
CD36	Forward	TCT​TTC​CTG​CAG​CCC​AAT​GGT​G
	Reverse	TGT​GAA​GTT​GTC​AGC​CTC​TGT​TCC
CD44	Forward	CAC​CGG​GCA​CTC​ACC​GAT​CTG​CGC​C
	Reverse	CCC​GTG​AGT​GGC​TAG​ACG​CGG​CAA​A
CD206	Forward	ATC​ACA​GAA​ATT​CCG​ATG​GGT​GTC
	Reverse	AGA​GAG​TGA​TAG​CAA​CCC​AGT​CC
TLR2	Forward	TGG​CAT​GTG​CTG​TGC​TCT​GTT​C
	Reverse	ATA​CCA​CAG​GCC​ATG​GAA​ACG​G
TLR4	Forward	TGT​TGT​GGT​GTC​CCA​GCA​CTT​C
	Reverse	ACT​GCC​AGG​TCT​GAG​CAA​TCT​C
RAGE	Forward	GAC​ATG​TGT​GTC​AGA​GGG​AAG​C
	Reverse	TTC​CCA​TCC​AAG​TGC​CAG​CTA​AG

### ELISA

The secretion of SASP (IL-6, IL-8, MMP-1 or MMP-3) was determined using an ELISA kit (Dakewei Biotech, Shenzhen, China). The HaCaT or HFF-1 cultures, both control or carnosine-treated, were centrifuged to obtain the culture supernatants, and the extent of SASP secretion in the supernatants was then determined according to the manufacturer’s instruction.

### Phagocytosis Assay

The localization of microspheres could be identified by preliminary confocal observation without the need to accurately quantify the extent of phagocytosis. Phagocytosis assay was performed using fluorescence microscopy to evaluate the effect of carnosine on the phagocytic activity of macrophages as previously described (Sharma et al., 2014). Briefly, Mφ cells were incubated for 24 h in a 12-well plate, followed by treatment with carnosine at different concentrations (5, 10 and 30 mM) for 24 h. After opsonization by mixing with FBS for 1 h, the FITC labeled microspheres were suspended in RPMI 1640 medium containing Mφ for 2 h. The plates were then placed on ice for 30 s to stop the phagocytosis. The non-phagocytosed beads were washed with PBS and then removed, and the fluorescence of the beads outside the cells was quenched by Trypan Blue solution (0.04%) for 30 min. After that, Mφ cells were fixed with 4% paraformaldehyde and then stained with rhodamine phalloidin (5 U/ml) for 2 h to mark the actin cytoskeleton. After washing with PBS, the fluorescence of the samples was measured using a Ti2-E&CSU-W1 confocal microscope (Nikon, Tokyo, Japan). The numbers of FITC positive cells was quantified with an ACEA NovoCyte flow cytometer (Agilent, Santa Clara, CA, United States), which enabled the number of Mφ cells to be quantified because they contained the phagocytosed microspheres. The number of Mφ cells was analyzed using NovoExpress software.

### Western Blot

THP-1 cells were cultured in a 6-well plate at a density of 5 × 10^4^ cells/well for 24 h and then induced with PMA for 6 h followed by treatment with carnosine at different concentrations. After that, the cells were collected, lysed with lysis buffer, and centrifuged at 14,000 ×*g* to collect the supernatant. The protein concentration in the supernatant was measured with a BCA protein assay kit (Pierce, Rockford, IL, United States). A sample of the supernatant containing 20 μg protein was subjected to SDS-PAGE using 12% gel. The proteins in the gel were then transferred to a piece of polyvinylidene difluoride (PVDF) membrane and blocked with 5% skim milk for 60 min. After that, the membrane was incubated with a 1:1,000 dilution of an appropriate primary antibody (anti-AKT2 (#2964), anti-phospho-AKT2 (#4060), anti-ERK1/2 (#4695), anti-phospho-ERK1/2 (#9101), anti-JNK (#9252), anti-phospho-JNK (#9251), anti-p38 (#9212), anti-phospho-p38 (#4511) or anti-Tubulin (#2146) (Cell Signaling Technology, Beverly, MA, United States) for overnight at 4 °C. This was followed by incubation with a secondary antibody (goat anti-rabbit or goat anti-mouse antibody) (Cell Signaling Technology, Beverly, MA, United States). Finally, the blot was visualized with a chemiluminescence substrate (Thermo scientific, NY, United States) and images of the blot were captured with an Amersham Imager (GE Healthcare Biosciences, Pittsburgh, PA, United States) and analyzed using the ImageJ software (U. S. National Institutes of Health, Bethesda, Maryland, United States).

### Statistical Analysis

All quantitative data were presented as means ± SD from at least three independent experiments. A comparison of multi-group data was carried out with a One-way analysis of variance (ANOVA) followed by Dunnett's test. Statistical significance was considered at either the *p* < 0.05 or *p* < 0.01 level. All graphical presentations were done using GraphPad Prism 5.01 (GraphPad, San Diego, CA, United States).

## Results

### tBHP Induces Senescent Skin Cells

**FIGURE 1:**
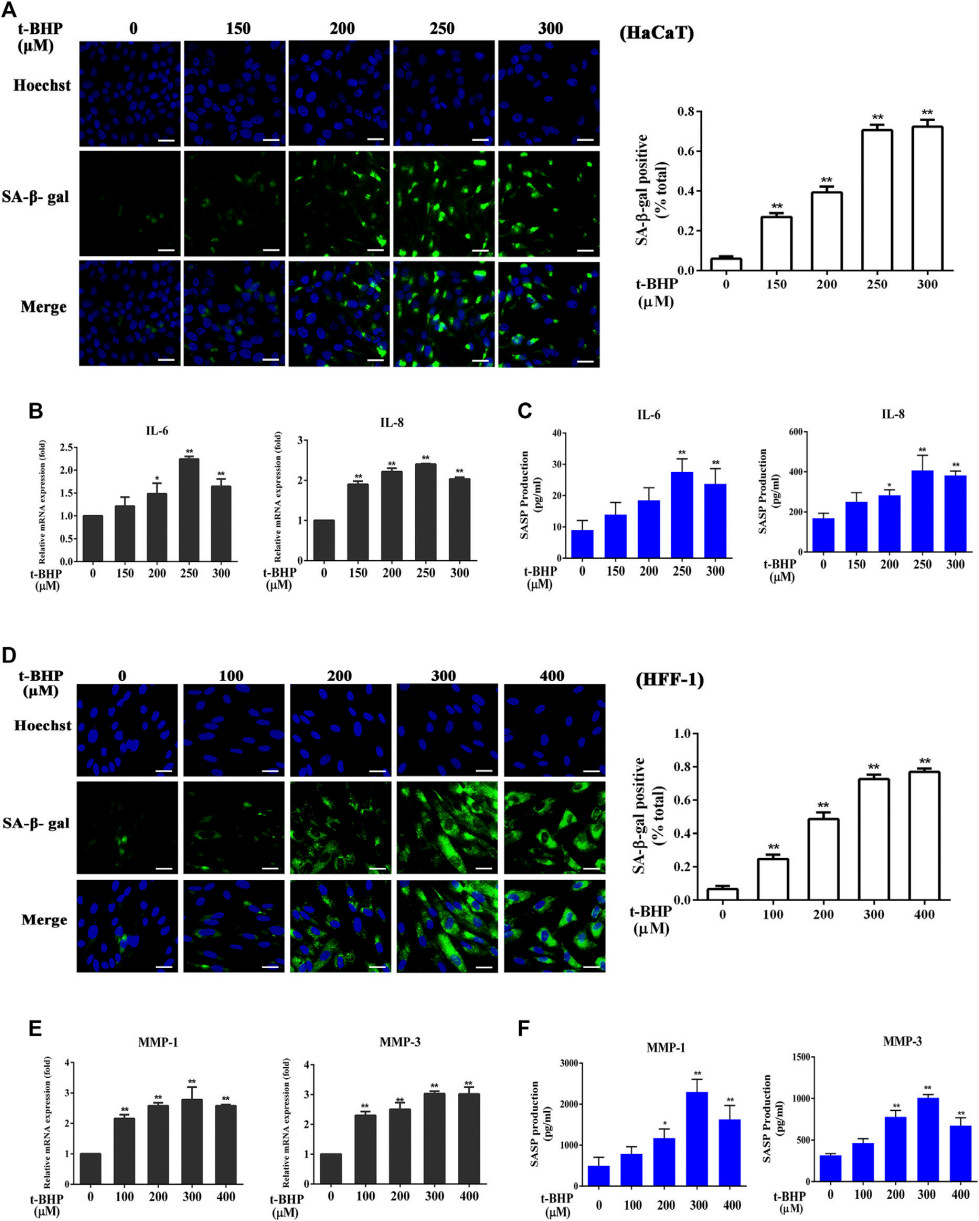
Induction of senescent HaCaT and HFF-1 cells by tBHP. **(A)** Representative image of tBHP-induced SA-β-Gal positive senescent HaCaT cells. The plot compares the percentage of SA-β-Gal positive cells. **(B)** Effect of tBHP on IL-6 and IL-8 mRNA levels in HaCaT cells as measured by *q*RT-PCR. **(C)** Effect of tBHP on IL-6 and IL-8 secretion by HaCaT cells as measured by ELISA. **(D)** Representative image of tBHP-induced SA-β-Gal positive senescent HFF-1 cells. The plot compares the percentage of SA-β-Gal positive cells. **(E)** Effect of tBHP on MMP-1 and MMP-3 mRNA levels in HFF-1 cells. **(F)** Effect of tBHP on MMP-1 and MMP-3 secretion by HFF-1 cells as measured by ELISA. Data are the means ± SD from three independent experiments. ‘*’ and ‘**’ indicate significantly different from the control at the *p* < 0.05 and *p* < 0.01 levels, respectively.

Incubation of HaCaT cells with tBHP at 150–300 μM for 2 h followed by 12 h incubation without tBHP significantly increased the number of SA-β-gal-positive HaCaT cells (Figure 1A). Such treatment also induced the expression of SASP factors, in particular, that of IL-6 and IL-8, both at the mRNA and protein levels (Figures 1B,C), indicating that tBHP could serve as a senescence inducer for HaCaT cells. Therefore, 250 μM of tBHP was chosen as the optimum concentration in subsequent experiments. tBHP also raised the percentage of SA-β-gal-positive HFF-1 cells (Figure 1D) and the mRNA and protein levels of matrix metalloproteinase 1 (MMP-1) and MMP-3 (Figures 1E,F), which peaked at 300 μM. Therefore, 300 μM tBHP was used in all subsequent experiments that involved HFF-1 cells.

### Preparation of the Macrophage (Mφ) and Senescent Skin Cell Co-culture

**FIGURE 2:**
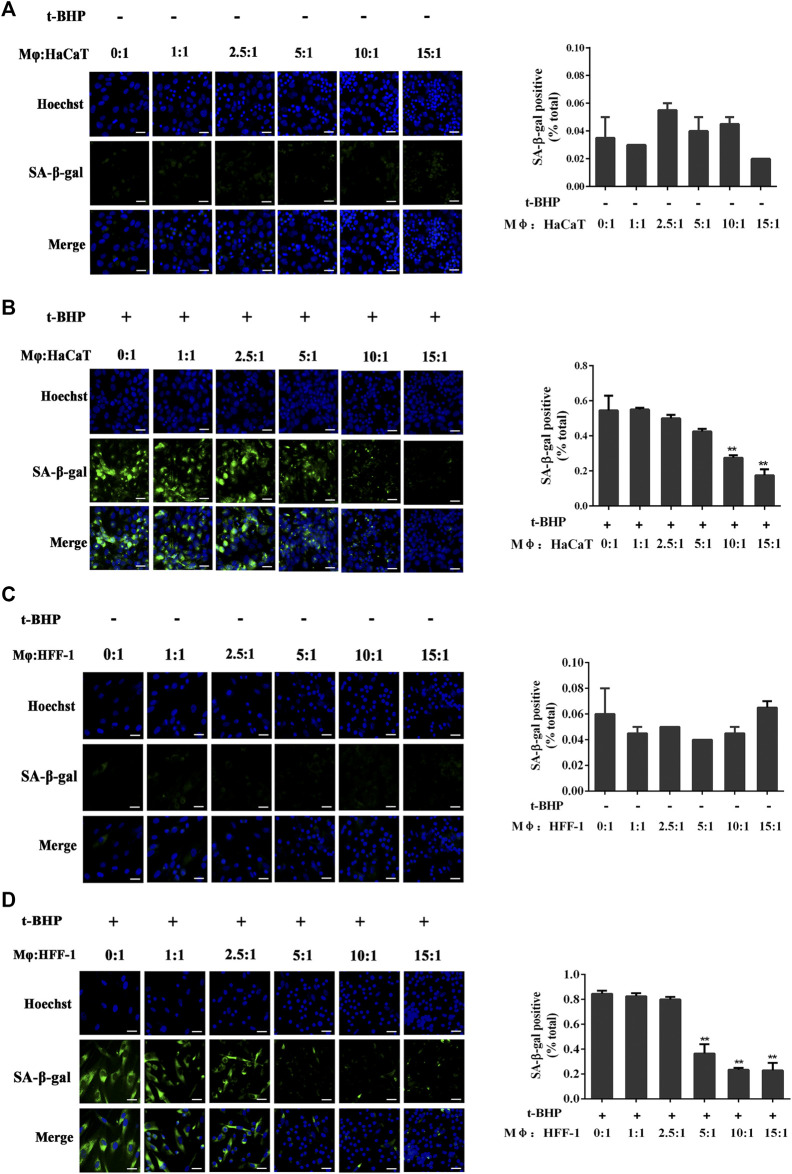
Co-culturing of THP-1-derived macrophages (Mφ) and senescent skin cells. **(A)** Representative image of SA-β-Gal positive senescent HaCaT cells in the Mφ-HaCaT co-culture. **(B)** Representative image of tBHP-induced SA-β-Gal positive senescent HaCaT cells in the Mφ-HaCaT co-culture. **(C)** Representative image of SA-β-Gal positive senescent HFF-1 cells in the Mφ-HFF-1 co-culture.**(D)** Representative image of tBHP-induced SA-β-Gal positive senescent HFF-1 cells in the Mφ-HFF-1 co-culture. The plots beside the images compare the percentage of SA-β-Gal positive cells. Data are the means ± SD from three independent experiments. ‘**’ indicates significantly different from the control at the *p* < 0.01 levels.

Mφ had no effect on non-senescent HaCaT (Figure 2A) or HFF-1 cells (Figure 2C). However, with tBHP-induced senescent HaCaT and HFF-1 cells, Mφ could reduce their number when co-cultured at ratios of 10:1 and 15:1 in the case of Mφ and HaCaT cells (Figure 2B) or 5:1, 10:1 and 15:1 for Mφ and HFF-1 cells (Figure 2D). The results clearly demonstrated the ability of macrophages to eliminate senescent HaCaT and HFF-1 cells from the co-cultures. A ratio 5:1 for Mφ to senescent cells was selected for subsequent co-cultures.

### Carnosine Improves the Ability of Macrophages to Remove Senescent Skin Cells

**FIGURE 3:**
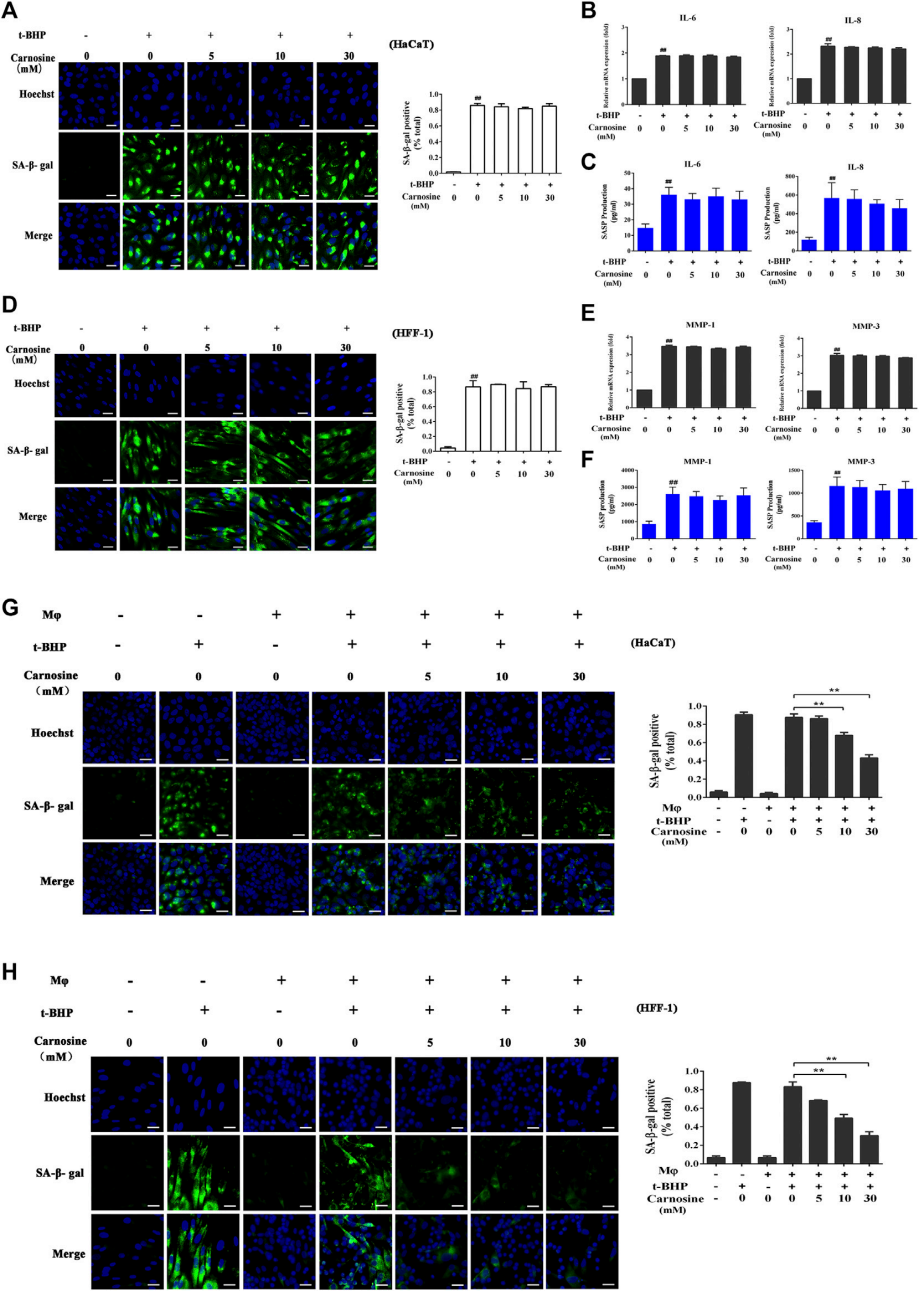
Effect of carnosine on senescent skin cells cultured alone or with Mφ. **(A)** Representative image of SA-β-Gal positive senescent HaCaT cells. **(B)** Effect of carnosine on IL-6 and IL-8 mRNA levels in tBHP-induced HaCaT cells. **(C)** Effect of carnosine on the secretion of IL-6 and IL-8 by tBHP-induced HaCaT cells. **(D)** Representative image of SA-β-Gal positive senescent HFF-1 cells. **(E)** Effect of carnosine on MMP-1 and MMP-3 mRNA levels in tBHP-induced HFF-1 cells. **(F)** Effect of carnosine on the secretion of MMP-1 and MMP-3 by tBHP-induced HFF-1 cells. **(G)** Representative image of tBHP-induced SA-β-Gal positive senescent HaCaT cells in the Mφ-HaCaT co-culture with and without carnosine. **(H)** Representative image of tBHP-induced SA-β-Gal positive senescent HFF-1 cells in the Mφ-HFF-1 co-culture with and without carnosine. The plots beside the images compare the percentage of SA-β-Gal positive cells. Data are the means ± SD from three independent experiments. ‘**’ indicates significantly different from the control at the *p* < 0.01 level.

Treatment of the skin cell monocultures with carnosine (5, 10, and 30 mM) neither decreased the number of SA-β-gal-positive HaCaT cells (Figure 3A) nor affected the expression of IL-6 and IL-8 (Figures 3B,C) expression. Carnosine also did not reduce the number of SA-β-gal-positive HFF-1 cells (Figure 3D) and lower the expression of MMP-1 and MMP-3 in these cells (Figures 3E,F). These results thus showed that treatment with carnosine could not reduce the number of senescent HaCaT and HFF-1 cells. In contrast, the application of 10 mM or 30 mM carnosine could reduce the number of senescent HaCaT (Figure 3G) and HFF-1 cells (Figure 3H) in the co-culture, suggesting that the presence of carnosine could facilitate the ability of macrophages to remove the senescent skin cells.

### Carnosine Elevates the Expression of CD36 and Receptor for Advanced Glycation End Products (RAGE) and Augments the Phagocytic Activity of Macrophages

**FIGURE 4:**
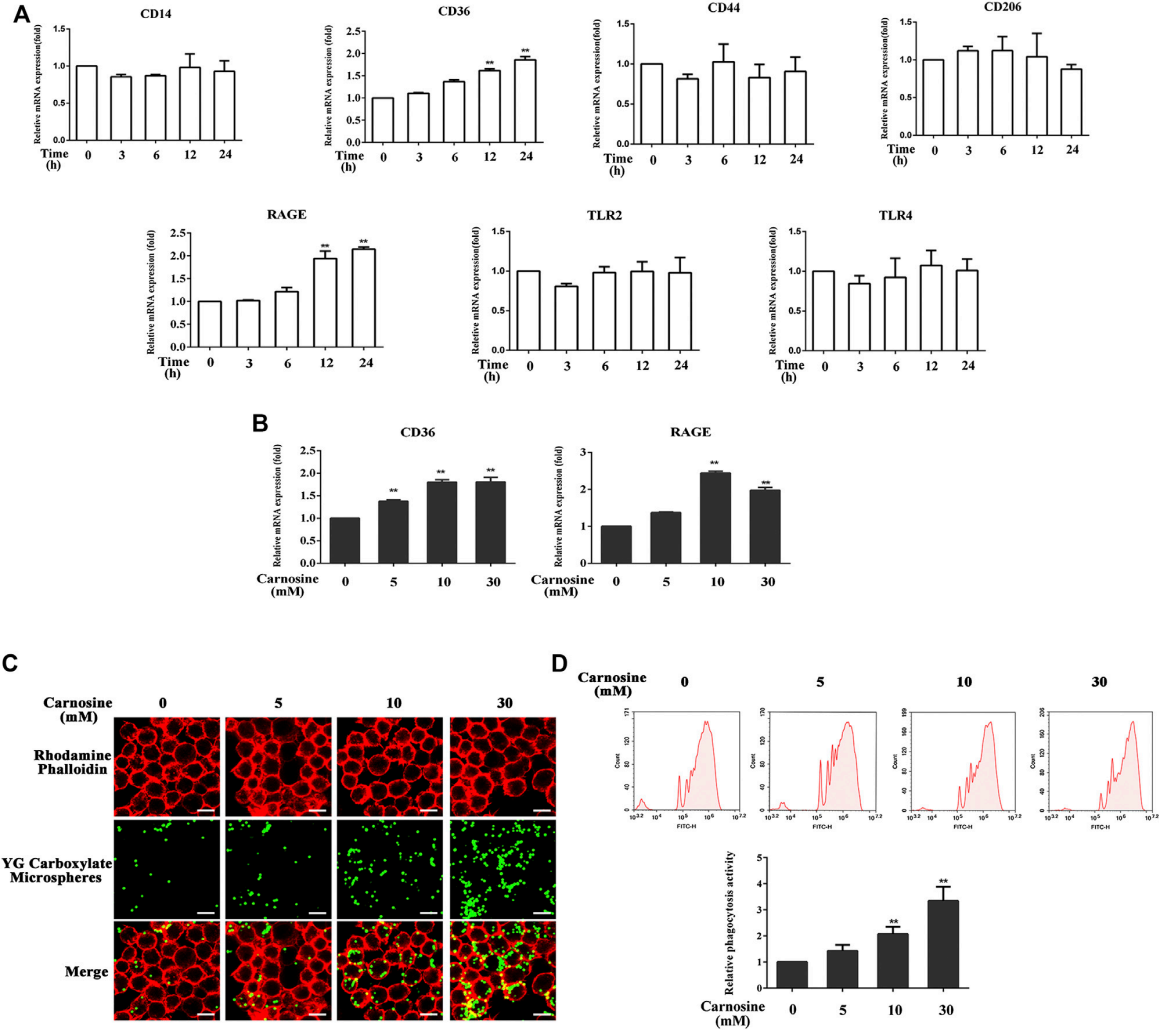
Effect of carnosine on the phagocytic activity of Mφ. **(A)** *q*RT-PCR analysis of CD14, CD36, CD44, CD206, RAGE, TLR2, TLR4 mRNA levels in Mφ following exposure to carnosine for the indicated time. **(B)** *q*RT-PCR analysis of CD36 and RAGE mRNA levels in Mφ exposed to 5, 10 or 30 mM carnosine for 12 h. **(C)** Phagocytosis activity of Mφ as observed by confocal microscopy. **(D)** Phagocytosis activity of Mφ as determined by flow cytometry. Data are the means ± SD from three independent experiments. ‘**’ indicates significantly different from the control group at the *p* < 0.01 levels.

The toxicity of carnosine on macrophages was evaluated by the CCK-8 assay. Treatment of macrophages with carnosine at 5, 10 and 30 mM for 24 h did not exert any significant cytotoxicity on the cells (data not shown). Macrophages express a variety of receptors involved in cell recognition and engulfment, including CD14, CD36, CD44, CD206, TLR2, TLR4 and RAGE (Hazen, 2008; Hart et al., 2012; Lee et al., 2018; Lu and Chen, 2019; Mohammadi et al., 2019; Duffney et al., 2020; Lee et al., 2020). After pretreatment of the macrophages with 30 mM carnosine for 0, 3, 6, 12 and 24 h, the levels of CD36 and RAGE mRNAs were significantly increased at 12 and 24 h, respectively, after the treatment (Figure 4A). Further investigation also demonstrated that all concentrations of carnosine tested could elevate the levels of CD36 and RAGE mRNAs at 12 h (Figure 4B). These results suggested that carnosine could augment the recognition ability of macrophages. Since phagocytosis is a primary process for removing pathogens and apoptotic cells (Underhill and Goodridge, 2012), the extent of phagocytosis by macrophages following treatment with carnosine was examined using a phagocytosis assay. The result clearly demonstrated that carnosine could enhance the phagocytic activity of macrophages (Figures 4C,D).

### Carnosine Promotes the Removal of Senescent Cells Through Elevated Expression of CD36 and RAGE

**FIGURE 5:**
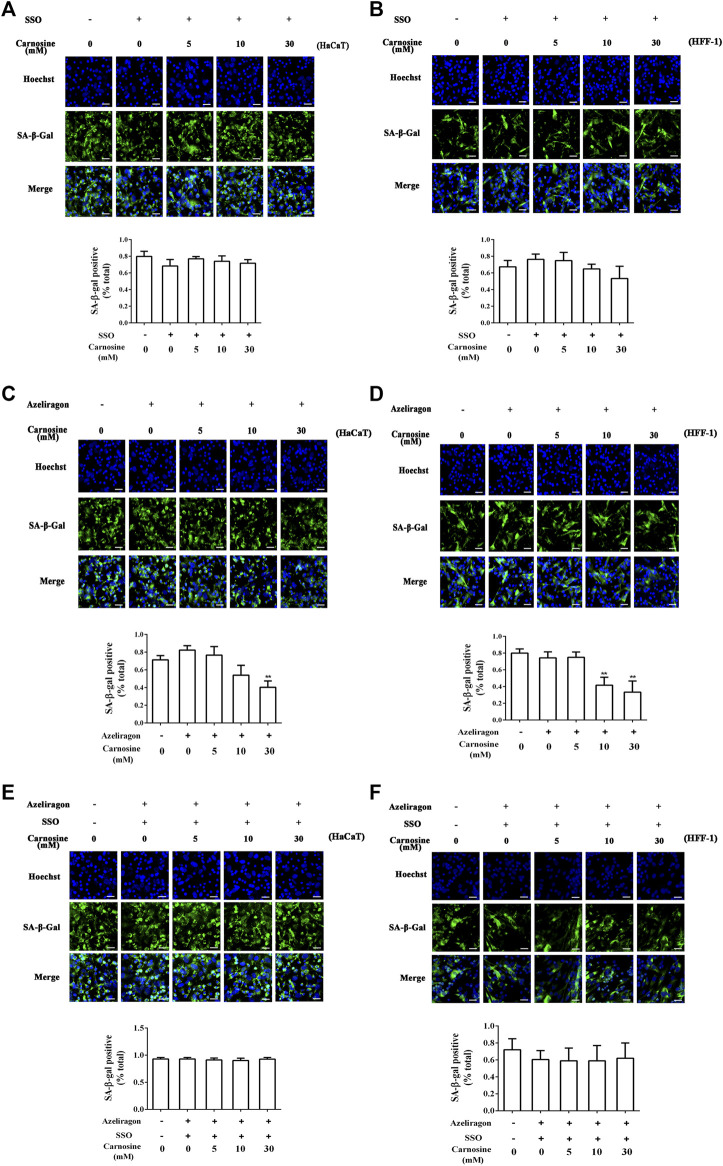
Effect of carnosine on the co-culture of senescent skin cells and Mφ pretreated with CD36 and RAGE antagonists. **(A)** Representative image of tBHP-induced SA-β-Gal positive senescent HaCaT cells in the Mφ-HaCaT co-culture treated with CD36 antagonists in the presence of carnosine. **(B)** Representative image of tBHP-induced SA-β-Gal positive senescent HFF-1 cells in the Mφ-HFF-1 co-culture treated with CD36 antagonists in the presence of carnosine. **(C)** Representative image of tBHP-induced SA-β-Gal positive senescent HaCaT cells in the Mφ-HaCaT co-culture treated with RAGE antagonists in the presence of carnosine. **(D)** Representative image of tBHP-induced SA-β-Gal positive senescent HFF-1 cells in the Mφ-HFF-1 co-culture treated with RAGE antagonists in the presence of carnosine. **(E)** Representative image of tBHP-induced SA-β-Gal positive senescent HaCaT cells in the Mφ-HaCaT co-culture treated with CD36 and RAGE antagonists in the presence of carnosine. **(F)** Representative image of the tBHP-induced SA-β-Gal positive senescent HFF-1 cells in the Mφ-HFF-1 co-culture treated with CD36 and RAGE antagonists in the presence of carnosine. The plots beside the images compare the percentage of SA-β-Gal positive cells. Data are the means ± SD from three independent experiments. ‘**’ indicates significantly different from the control group at the *p* < 0.01 level.

The effects of CD36 and RAGE antagonists on the ability of macrophages to eradicate senescent skin cells in the presence of carnosine was investigated. Treatment of macrophages with sulfosuccinimidyl oleate sodium (SSO, a CD36 antagonist) or azeliragon (a RAGE antagonist) failed to enhance the ability of the macrophages to clear the senescent HaCaT (Figures 5A,C) and HFF-1 cells (Figures 5B,D) from the co-culture in the presence of carnosine. However, in the case of RAGE antagonist, clearance of HcCaT cells was still detectable in the presence of 30 mM carnosine, while both 10 and 30 mM carnosine could still result in enhanced clearance of HFF-1 cells. Treatment of macrophages with both SSO and azeliragon also did not increase the ability of the macrophages to clear the senescent HaCaT or HFF-1 cells from the co-culture (Figures 5E,F). These data, therefore, indicated that the ability of carnosine to enhance the ability of macrophages to remove the senescent skin cells could be suppressed when the action of CD36 or RAGE was blocked by an inhibitor, although blocking the activity of RAGE had a lesser effect compared with the blockage of CD36 activity. Taken together, these results demonstrated that carnosine promoted the removal of senescent cells through elevated expression of CD36 and RAGE.

### Carnosine Activates the AKT2 Signaling Pathway

**FIGURE 6:**
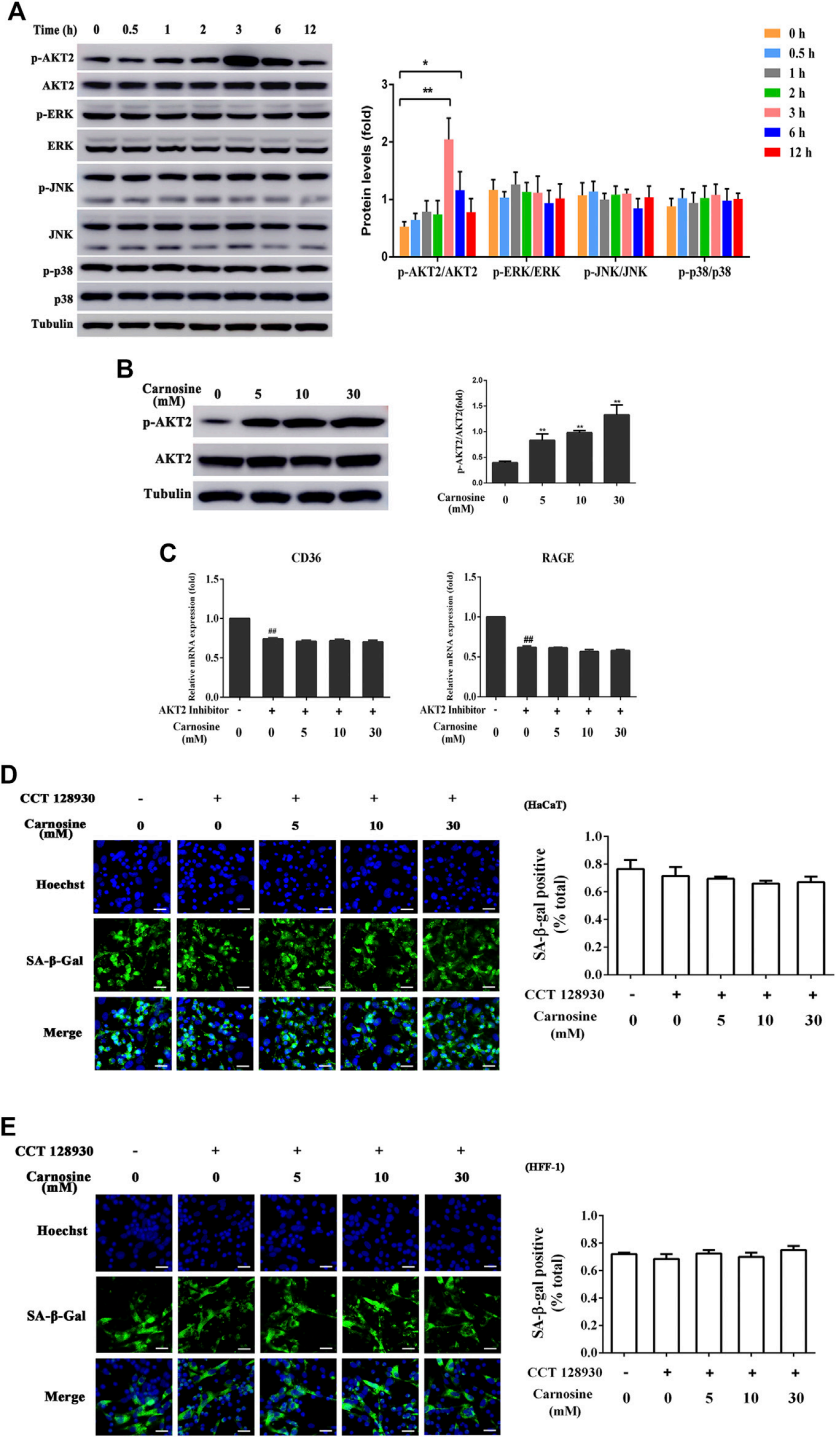
Effect of carnosine on the AKT2 and MAPK signaling pathway. **(A)** Western blot analysis of the levels of AKT2, phospho-AKT2, ERK, phospho-ERK, JNK, phospho-JNK, p38, phospho-p38 in Mφ cells following treatment without or with 30 mM carnosine. **(B)** Western blot analysis of the levels of AKT2, phospho-AKT2 in Mφ cells following treatment without or with carnosine at the indicated concentrations. **(C)** *q*RT-PCR analysis of CD36 and RAGE mRNA levels in HaCaT following treatment with carnosine without or with 10 mM CCT128930 (AKT two inhibitor). **(D)** Representative image of the tBHP-induced SA-β-Gal positive senescent HaCaT cells in the Mφ-HaCaT co-culture treated with CCT128930 in the presence of carnosine. **(E)** Representative image of the tBHP-induced SA-β-Gal positive senescent HFF-1 cells in the Mφ-HFF-1 co-culture treated with CCT128930 in the presence of carnosine. Data are the means ± SD from three independent experiments. ‘*’ and ‘**’ indicate significantly different from the control group at the *p* < 0.05 and *p* < 0.01 levels, respectively.

To further probe the related intracellular mechanisms of carnosine, its effect on the activation of the AKT2 and MAPK pathways was investigated. Carnosine induced significant phosphorylation of AKT2 in the macrophages at 3 and 6 h after treatment but showed no effect on the activation of MAPK (ERK, JNK and p38) signaling pathway (Figure 6A). Treatment of macrophages with carnosine at 5, 10 and 30 mM for 3 h resulted in the activation of the AKT2 signaling pathway in a dose-dependent manner (Figure 6B). Treatment of macrophages with carnosine in the absence or presence of CCT128930 (an AKT2 antagonist) for 2 h led to the attenuation of CD36 and RAGE expression (Figure 6C). These results suggested that carnosine may increase the recognition of senescent skin cells by macrophages and the phagocytic activity of macrophages by activating the AKT2 pathway. In addition, the ability of macrophages to reduce senescent HaCaT (Figure 6D) and HFF-1 cells (Figure 6E) was further blocked by the AKT2 antagonist, indicating that carnosine may elevate the senescent cell-clearance capacity of macrophages through activation of the AKT2 signaling pathway.

## Discussion

**FIGURE 7:**
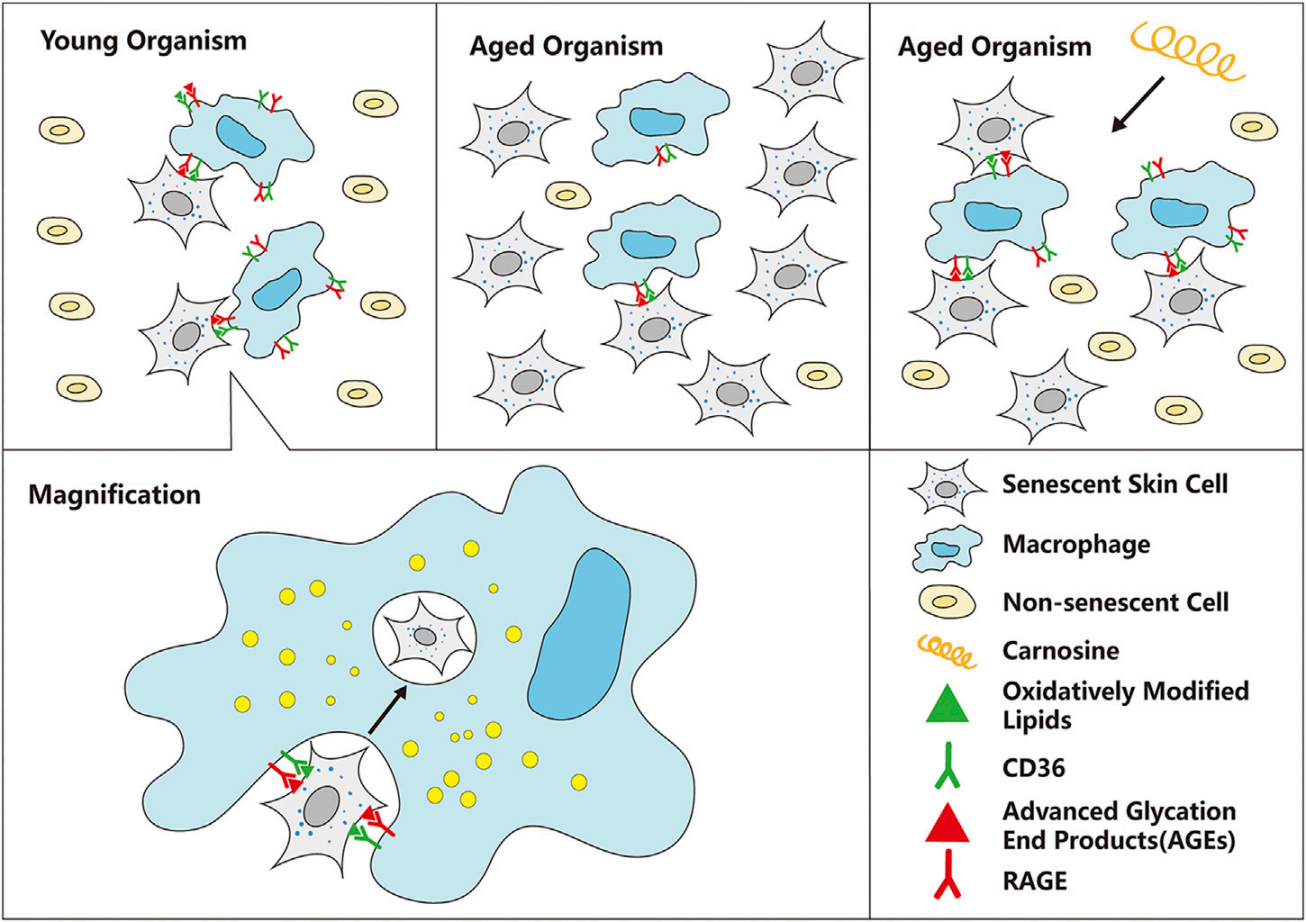
Schematic diagram of the mechanism by which carnosine stimulates macrophages to eliminate senescent skin cells. Macrophages can selectively recognize and eliminate senescent skin cells mainly through the actions of CD36 and RAGE, but this function is impaired with age, resulting in excessive accumulation of senescent cells in the skin. Carnosine can up-regulate the expression of CD36 and RAGE and improve the phagocytosis ability of macrophages, thus improving the ability of macrophages to eliminate senescent skin cells.

We have established a cell culture model in which macrophages were co-cultured with senescent skin cells to study the ability of the macrophages to eliminate the senescent skin cells from the culture. Two types of skin cells were used in this co-culture model, HaCaT and HFF-1 cells, which are keratinocytes and fibroblasts, respectively. Subsequent experiments showed that the addition of carnosine (a dipeptide that is synthesized endogenously) to the co-culture could result in the enhanced clearance of both senescent HaCaT and HFF-1 cells. Further mechanistic study showed that carnosine could upregulate the expression of CD36 and RAGE through activating the AKT2 signaling pathway as well as elevating the phagocytic ability of the macrophages. Both mechanisms appeared to promote the ability of the macrophages to eliminate the senescent skin cells, possibly through phagocytosis (Figure 7).

The excessive accumulation of senescent skin cells can lead to the onset and progression of skin aging (Burton and Krizhanovsky, 2014; Sharpless and Sherr, 2015; Hernandez-Segura et al., 2018). Tert-butyl hydroperoxide (tBHP) is a known inducer of oxidative stress and its stimulating effect on skin aging has previously been demonstrated (Wedel et al., 2020). As an organic peroxide, tBHP is either metabolized by cytochrome P450 in the cells to generate peroxyl and alkoxyl radicals or detoxified to tert-butanol, both of which can inflict rapid oxidative damage to the cells, eventually resulting in cell senescence. Thus, tBHP was employed to induce senescence in HaCaT and HFF-1 in this study. A number of tBHP concentrations were tested, and 250–300 μM appeared to stimulate the maximum extent of senescence (Figure 1), similar to those reported in a previous study (Ding et al., 2018).

The elimination of senescent cells could serve as a therapeutic approach to reverse the process of aging in humans. Existing therapeutic strategies for preventing and eliminating senescent cells include 1) improvement of the immune system, 2) selective induction of cell death, and 3) inhibition of SASP (Velarde and Demaria, 2016; Gruber et al., 2020). The application of compounds that can induce senescent cell death (also known as ‘senolytics’) is specifically limited because of the intrinsic toxicity of these compounds. Moreover, the use of SASP inhibitors (which have been used as anti-inflammatory drugs) to suppress senescence is also limited because of their non-specificity, resulting in severe toxicity induced by long-term use (Soto-Gamez and Demaria, 2017; Hernandez-Segura et al., 2018). SASP factors tend to play a central role in the detection of senescent cells and their clearance from the skin mediated by the immune cells (Ghosh and Capell, 2016). Secreted proinflammatory cytokines and chemokines are the key elements of inflammation, which can attract immune cells, while the extracellular matrix facilitates the entry of immune cells to the inflamed areas to eliminate the senescent cells, a mechanism that is well regulated (Borodkina et al., 2018). However, in aged individuals, the immune cells may also age by entering a stage known as immunosenescence, and this will reduce their ability to eliminate senescent cells, thereby exacerbating the stage of inflammation (Effros and Pawelec, 1997). Consequently, approaches for restoring the aging immune system may be one of the safer strategies for eliminating senescent cells. Previous studies have proposed that macrophages play a critical role in the immune surveillance of senescent cells. A recent study has shown that alginate beads containing senescent cells can promote the recruitment of macrophages when transplanted into mice (Hall et al., 2016). Our data explicitly showed that THP-1 derived macrophages could only eliminate senescent HaCaT and HFF-1 cells in the co-culture while having no effect on non-senescent HaCaT and HFF-1 cells (Figure 2). Although carnosine could promote the clearance of senescent cells by macrophages, it had no effect on the number of senescent HaCaT and HFF-1 cells in the absence of macrophages (Figure 3).

Further investigation demonstrated that carnosine could elevate the expression of CD36 and RAGE in macrophages (Figures 4A,B). RAGE and CD36 play a critical role in the recognition and subsequent phagocytosis of the senescent cells by macrophages. RAGE is expressed by different types of dermal cells, including macrophages, monocytes and T lymphocytes, and it has been shown to enhance macrophage-mediated recognition and clearance of apoptotic cells (Yan et al., 2010; Pageon et al., 2017). However, RAGE can bind to advanced glycosylation end-products (AGEs), which are non-enzymatical glycosylated proteins that play a critical role in the removal of senescent cells in the aging process. The binding of AGEs to RAGE would therefore incapacitate its ability to stimulate macrophage-mediated recognition and clearance of apoptotic cells. Oxidatively modified lipids on the cell membranes can be used as a pattern recognition ligand to promote the recognition and subsequent phagocytosis of apoptotic cells by macrophages through the scavenger receptor CD36 (Friggeri et al. 2011). The result obtained from phagocytosis assays was consistent with the observation that carnosine could improve the ability of macrophages to remove senescent skin cells (Figures 4C,D). Furthermore, both CD36 and RAGE inhibitors effectively suppressed the stimulating effect of carnosine on the clearance of senescent HaCaT and HFF-1 cells mediated by macrophages.

Several previous studies have also shown that the MAPK family plays a critical role in the regulation of phagocytosis (Downey et al., 1998; Schnyder et al., 1998; Zelck et al., 2007). Macrophages predominantly express AKT1 and AKT2 but not AKT3. AKT2 controls phagocytosis through multiple downstream targets, such as mTOR and p70S6K (Shiratsuchi and Basson, 2007; Weichhart and Säemann, 2008). According to our data, when the AKT2 pathway was blocked by the use of CCT128930, the inclusion of carnosine in the co-culture did not result in any detectable increase in CD36 and RAGE mRNA levels (Figures 6D,E), suggesting that the enhancing effect of carnosine on macrophage with respect to the clearance of senescent cells might involve the activation of the AKT2 pathway. However, whether such an effect of carnosine will also be observed *in vivo* is a subject of further research.

## Conclusion

A co-culture model consisting of macrophages and a type of senescent skin cell was used to establish the anti-aging effect of carnosine. In this model, carnosine was found to promote the clearance of senescent skin cells by enhancing their phagocytosis mediated by the macrophages. The underlying mechanism of this effect appeared to involve the upregulation of CD36 and RAGE expression, presumably stimulated by carnosine via the activation of the AKT2 signaling pathway.

## Data Availability Statement

The raw data supporting the conclusions of this article will be made available by the authors, without undue reservation, to any qualified researcher.

## Author Contributions

NX and ZH conceived and designed the experiments. XL, KY and SG performed the experiments. JZ and GL analyzed the data. YC, HL and WZ prepared the manuscript. ZH and NX revised the manuscript.

## Funding

The work was supported by National Natural Science Foundation of China (81903736) and Scientific and Technological Plan Project of Wenzhou (N20180002).

## Conflict of Interest

Author KY and GL were employed by the Infinitus (China) Company.

The remaining authors declare that the research was conducted in the absence of any commercial or financial relationships that could be construed as a potential conflict of interest.
